# Is conservative treatment always safe in unifocal clinically T1a/node‐negative papillary thyroid carcinoma?

**DOI:** 10.1002/wjs.12440

**Published:** 2024-12-04

**Authors:** Francesco Pennestrì, Priscilla Francesca Procopio, Antonio Laurino, Annamaria Martullo, Gloria Santoro, Pierpaolo Gallucci, Francesca Prioli, Luca Sessa, Esther Diana Rossi, Alfredo Pontecorvi, Carmela De Crea, Marco Raffaelli

**Affiliations:** ^1^ UOC Chirurgia Endocrina e Metabolica Fondazione Policlinico Universitario Agostino Gemelli IRCCS Rome Italy; ^2^ Centro di Ricerca in Chirurgia delle Ghiandole Endocrine e dell’Obesità Università Cattolica del Sacro Cuore Rome Italy; ^3^ Dipartimento di Scienze Mediche e Chirurgiche Addominali ed Endocrino‐Metaboliche Fondazione Policlinico Universitario Agostino Gemelli IRCCS Rome Italy; ^4^ Fondazione Istituto G. Giglio Cefalù Palermo Italy; ^5^ UniCamillus Saint Camillus International University of Health and Medical Sciences Rome Italy; ^6^ UOC Anatomia Patologica della Testa e Collo, del Polmone e dell’Apparato Endocrino Fondazione Policlinico Universitario Agostino Gemelli IRCCS Rome Italy; ^7^ Dipartimento di Scienza della Vita e Sanità Pubblica Università Cattolica del Sacro Cuore Rome Italy; ^8^ Medicina Interna, Endocrinologia e Diabetologia Fondazione Policlinico Universitario Agostino Gemelli IRCCS Rome Italy; ^9^ Dipartimento di Medicina e Chirurgia Traslazionale Università Cattolica del Sacro Cuore Rome Italy

**Keywords:** frozen section examination (FSE), LVI, lymph node metastases, papillary thyroid cancer, PTMC, young age

## Abstract

**Background:**

Management of clinically unifocal node‐negative papillary thyroid carcinoma ≤1 cm (PTMC) is controversial with nonsurgical treatment as a potential alternative to thyroid lobectomy (TL). However, conservative strategies, such as active surveillance or thermal ablation, do not allow the evaluation of biological aggressive features or occult lymph node metastases (LNMs), which play a primary role as prognostic factors.

**Methods:**

Among 4216 thyroidectomies for malignancy (between September 2014 and September 2023), TL plus ipsilateral central neck dissection was performed in 203 (4.8%) unifocal N0 PTMCs. Completion thyroidectomy was accomplished in case of positive frozen section examination of removed nodes or within 6 months from index operation in presence of biological aggressive features.

**Results:**

Seventy‐six out of 203 (37.4%) patients were staged pN1a and extranodal extension was detected in 5 (6.6%) patients. At final histology, biological aggressive features, including multifocality, lymphovascular invasion (LVI), extracapsular invasion, tumor aggressive subtypes, and BRAF‐V600E mutation, were detected in 69 (34%), 93 (45.8%), 3 (1.5%), 30 (14.8%), and 7 (3.5%) patients, respectively. A comparative analysis between pN0 and pN1a patients showed younger age (*p* < 0.001), LVI (*p* = 0.037), and multifocality (*p* < 0.001) as risk factors for occult central LNMs. After logistic regression analysis, age (*p* < 0.001) and multifocality (*p* < 0.001) were confirmed as independent risk factors for nodal involvement.

**Conclusions:**

Although most PTMC has been widely defined as indolent disease, a non‐negligible rate of patients may present one or more biologically aggressive features including nodal involvement. Nonsurgical management should be considered with caution to avoid undertreatment especially in the younger population.

## Graphical abstract

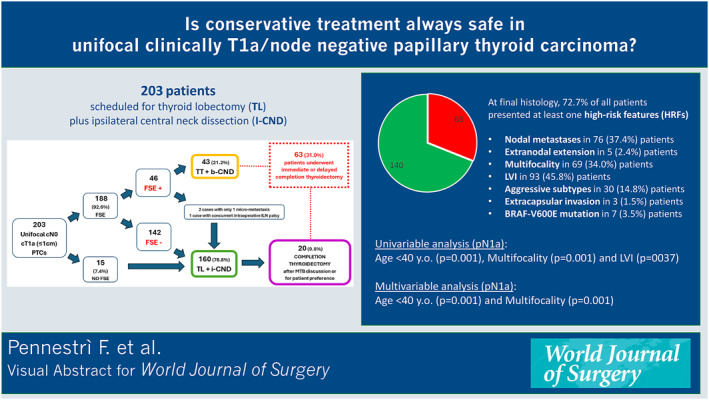

## 1 INTRODUCTION

Over the last few decades, a progressive increase in the incidence of differentiated thyroid cancer has been reported with papillary thyroid carcinoma (PTC) ≤1 cm (PTMCs) being mainly involved.^1^ As a consequence of this evidence and of PTMCs slow growth rate and relative indolent course,^2^, ^3^ less aggressive therapeutic strategies have been proposed.^4^, ^5^, ^6^ The 2015 American Thyroid Association (ATA) guidelines^7^ suggest thyroid lobectomy (TL) or active surveillance (AS) for low‐risk PTMCs in the absence of clinically detectable cervical lymph node metastases (LNMs) or other biological aggressive factors.

Although the recurrence rates are relatively low^8^ and most studies demonstrated that significant morbidity and mortality did not occur in patients under AS in the short run, PTMC is not completely innocuous.^9^ Delayed treatment of differentiated thyroid cancer for at least 12 months may increase the likelihood of cancer‐related death by 130%.^10^ In contrast to the more favorable long‐term prognosis of PTC, the incidence of LNMs in clinically node‐negative (cN0) PTMCs has been reported in up to 42% of cases.^11^, ^12^, ^13^

To date, no well‐defined preoperative clinical parameter is a clear predictor for central LNMs in cN0 clinically unifocal PTCs.^14^

Prophylactic (p) central neck dissection (CND) remains controversial in these patients' cohort because of the potential postoperative complications.^15^ Nevertheless, the incidence of central LNMs in cN0‐PTMCs is not low enough to ignore the necessity of p‐CND.^15^

Moreover, recent studies reported the central neck LNMs as a potential marker of aggressive biological behavior and a risk factor for recurrence, distant metastases, reduced survival, and higher morbidity.^16^, ^17^, ^18^, ^19^

As the presence of LNMs is the only risk factor that may be investigated during the index operation, intraoperative evaluation of nodal status by means of frozen section examination (FSE) may play a pivotal role in surgical decision‐making. We aimed to determine if the routine evaluation of lymph node status has relevant implications for risk stratification of clinically unifocal cN0‐PTMCs scheduled for TL.

## 2 MATERIAL AND METHODS

### 2.1 Study population

Among 4216 thyroidectomies for malignancy between September 2014 and September 2023 in a national referral center for endocrine surgery, 203 patients were scheduled for TL plus ipsilateral CND (i‐CND) for unifocal cT1aN0 PTCs. Informed consent concerning the risks and benefits of TL and total thyroidectomy (TT), basing on available guidelines,^7^, ^20^ was administrated to every patient.

Adult patients, classic PTCs and subtypes, clinically unifocal and intrathyroidal PTCs, clinical tumor size ≤1 cm, and cN0 PTCs were included. Exclusion criteria consisted in prior head/neck irradiation, family history of thyroid carcinoma, clinical evidence of multifocality, evidence of LNMs, and <6 months‐follow‐up. All data have been retrospectively collected in a prospective designed database for endocrine neoplasm. The study was approved by the Ethical Committee of our Center (ID 6584). The follow‐up ended on April 30^th^, 2024.

### 2.2 Definitions

CN0, TL, TT, i‐CND, and bilateral CND (b‐CND) have been previously defined and described in detail.^21^, ^22^, ^23^, ^24^, ^25^ All surgical procedures were performed by experienced endocrine surgeons or by young endocrine surgeons under supervision.^26^ Pathological tumor staging and PTC subtypes in terms of aggressive variants were redefined in accordance with the 8^th^ Edition of the AJCC/TNM staging system^27^ and the 2022 WHO classification.^28^ Micrometastases and macrometastases were defined based on ≤2 and >2 mm, respectively.^7^ Biological aggressive features included LNMs, extranodal extension, aggressive histology subtypes, lymphovascular invasion (LVI), extracapsular invasion, multifocality, and BRAF‐V600E mutation. PTC with aggressive subtype ≥30% or with infiltrative follicular variant were considered aggressive histological variants. Definition of transient/permanent nerve palsy^29^ and transient^30^/persistent^31^ hypoparathyroidism has been previously reported. Biochemical cure and persistent (biochemical and structural) disease were considered in accordance with ATA guidelines.^7^ Locoregional recurrence was defined as a clinically detected disease in the thyroid bed and/or in the central/lateral compartments.

### 2.3 Study design

TL plus i‐CND was performed as the first step of the procedure. Removed lymph nodes were sent for FSE if the pathologist was available. When ipsilateral occult LNMs were found on FSE, completion thyroidectomy (CT) plus completion CND was accomplished during the same procedure. Following multidisciplinary tumor board (MTB) discussion, in presence of biological aggressive features and/or based on patient's preference, CT plus completion CND was proposed within 6 months from the index operation.

For each included patient, demographic, perioperative, and follow‐up data were registered in a specifically designed deidentified database. Follow‐up data were obtained by outpatient consultations, medical charts, and/or telephone contact.

### 2.4 Preoperative assessment

Preoperative assessment encompassed physical examination, biochemical profile, fine‐needle aspiration biopsy, and laryngoscopy. Preoperative ultrasound was performed by an experienced surgeon in all patients to confirm preoperative staging.

### 2.5 Postoperative management and follow‐up evaluation

Patients who underwent TT plus b‐CND received radioactive iodine (RAI) based on the stage and risk factor evaluation.^7^, ^32^

### 2.6 Study endpoints

The primary endpoint was to assess the LNMs rate in patients with clinically unifocal cT1aN0 PTC. Secondary endpoints were the assessment of the rate of further biologically aggressive features rather than LNMs and the identification of potential risk factors for occult central LNMs.

### 2.7 Statistical analysis

Data were described through the median with interquartile ranges for numerical data through absolute frequency and percentage in case of categorical variables. Receiver operating characteristic (ROC) curve analysis has been used to assess the correlation between age and LNMs. The cutoff values with the best sensitivity and specificity for the detection of LNMs were calculated using Youden's index [sensitivity − (1 − specificity)]. The ROC curve analysis results were reported as the area under the curve (AUC) with corresponding *p*‐values. Univariable and multivariable analyses determined disease odds ratios for pN status. A significance level ≤0.05 allowed us to discriminate the conditions associated with the reference outcome. All statistical analyses were performed with the open‐source statistical software R (version 4.3.2).

## 3 RESULTS

Demographic, perioperative, and follow‐up characteristics of the included patients are reported in Table 1. FSE was not performed in 15 (7.4%) patients due to the unavailability of the pathologist. FSE identified occult central LNMs in 46 out of 188 patients. Forty‐three (22.9%) patients underwent TT plus b‐CND during the same operation; in 2 patients, FSE identified only 1 micrometastases and the first step remained TL plus i‐CND; in the remaining patient, one macrometastases was identified by FSE but CT was not performed due to the concurrent intraoperative inferior laryngeal nerve (ILN) loss of signal.

**TABLE 1:** Demographic, clinical, operative, pathologic, and follow‐up characteristics of the 203 included patients.

Age, years (median) (IQR)	42 (33–49)
Sex^a^ (%)	F: 156 (76.8%) / M: 47 (23.2%)
Preoperative tumor size, mm (median) (IQR)	6 (4–7)
FSE (%)	Negative: 142 (69.9%) / positive: 46 (22.7%) / NA: 15 (7.4%)
No. of LN on FSE (median) (IQR)	4 (3–5)
No. of LNM on FSE (median) (IQR)	1.5 (1–2)
Postoperative tumor size, mm (median) (IQR)	6 (5–8)
pN stage (%)	pN0: 127 (62.6%) / pN1a: 76 (37.4%)
No. of LN on definitive histology (median) (IQR)	7 (5–11)
No. of LNM on definitive histology (median) (IQR)	2 (1–3)
Extranodal extension (%)	5 (6.6%) out of 76 pN1a patients
Aggressive subtypes (%)	No: 173 (85.2%) / yes: 30 (14.8%)
Extracapsular invasion (%)	No: 200 (98.5%) / yes: 3 (1.5%)
LVI (%)	No: 110 (54.2%) / yes: 93 (45.8%)
Multifocality (%)	No: 134 (66.0%) / yes: 69 (34.0%)
BRAF‐V600E mutation (%)	No: 196 (96.5%) / yes: 7 (3.5%)
Transient ILN palsy (%)	18 (6.8%) out of 266 nerves at risk
Permanent ILN palsy (%)	2 (0.7%) out of 266 nerves at risk
Transient hypoparathyroidism (%)	20 (31.7%) out of 63 patients underwent immediate or delayed CT
Permanent hypoparathyroidism (%)	4 (6.3%) out of 63 patients underwent immediate or delayed CT
RAI (%)	34 (53.9%) out of 63 patients underwent immediate or delayed CT
Recurrence (%)	0 (0.0%)

Abbreviations: CT, completion thyroidectomy; F, female; FSE, frozen section examination; ILN, inferior laryngeal nerve; IQR, interquartile range; LN, lymph node; LNM, lymph node metastasis; LVI, lymphovascular invasion; M, male; and RAI, radioactive iodine.

^a^ As assigned at birth.

Overall, at final histology, 76 out of 203 (37.4%) patients were staged pN1a (with 37 patients with only micrometastases). Extranodal extension was observed in five cases. FSE detected false positive results in two patients with discordant results after definitive histopathology (pN0). LNMs were observed in 31 out of 142 patients in whom FSE was negative for nodal involvement (false negative results). In the patient cohort which did not receive FSE, one patient was staged pN1a after definitive histopathology.

After the index operation, 69 (34%) PTMC were multifocal and LVI was found in 93 (45.8%) patients. Extracapsular invasion was detected in 3 (1.5%) cases. There were 97 classic PTCs, 8 follicular variant PTCs, 67 PTCs with subtypes <30%, and 30 (14.8%) aggressive subtypes PTCs (21 infiltrative follicular PTCs, 8 tall cell PTCs, and 1 solid/trabecular PTCs). BRAF‐V600E mutation was found in 7 (3.5%) patients.

Among the 43 patients who underwent TT plus b‐CND for occult LNMs, final histology showed 11 bilateral multifocality and 17 bilateral central LNMs.

After MTB discussion, 20 additional patients out of 160 patients underwent delayed CT. In this patients' cohort, final histology showed contralateral PTCs foci in 6 cases and contralateral LNMs in 3 cases (Table 2).

**TABLE 2:** Demographic, clinical, and pathologic characteristics of the 63 patients who underwent to immediate or delay total thyroidectomy and bilateral central neck dissection.

Age, years (median) (IQR)	36 (29–44)
Sex^a^ (%)	F: 12 (19%) / M: 51 (81%)
Preoperative tumor size, mm (median) (IQR)	8 (7–9)
Postoperative tumor size, mm (median) (IQR)	6 (5–8)
pN stage (%)	pN0: 4 (6.4%) / pN1a: 59 (93.6%)
No. of LN on definitive histology (median) (IQR)	10 (6–14)
No. of LNM on definitive histology (median) (IQR)	2 (2–3)
Extranodal extension (%)	5 (8.5%) out of 59 pN1a patients
Bilateral central compartments LNM on definitive histology (%)	20 (33.9%) out of 59 pN1a patients
Aggressive subtypes (%)	No: 51 (81%) / yes: 12 (19%)
Extracapsular invasion (%)	No: 62 (98.4%) / yes: 1 (1.6%)
LVI (%)	No: 22 (44.9%) / yes: 41 (65.1%)
Multifocality (%)	No: 19 (30.2%) / yes: 44 (69.8%)
Bilateral multifocality (%)	17 (38.6%) out of 44 patients with multifocality
BRAF‐V600E mutation (%)	No: 62 (98.4%) / yes: 1 (1.6%)

Abbreviations: F, female; IQR, interquartile range; LN, lymph node; LNM, lymph node metastasis; LVI, lymphovascular invasion; and M, male.

^a^ As assigned at birth.

**FIGURE 1:**
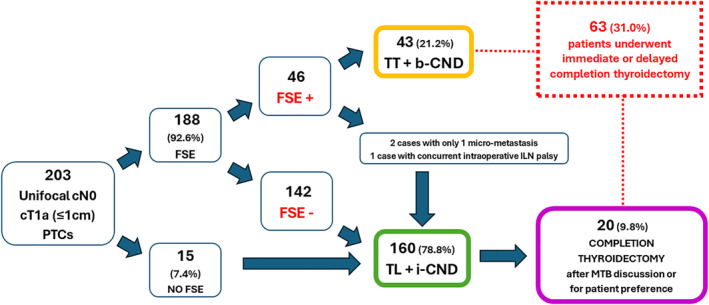
Treatment algorithm. b‐CND, bilateral central neck dissection; FSE, frozen section examination; i‐CND, ipsilateral central neck dissection; ILN, inferior laryngeal nerve; MTB, multidisciplinary tumor board; PTC, papillary thyroid cancer; TL, thyroid lobectomy; and TT, total thyroidectomy.

Figure 1 summarizes the treatment algorithm.

Overall, only 56 (27.6%) patients did not show aggressive biological features.

Transient and permanent hypoparathyroidism was observed in 20 (31.7%) and 4 (6.3%) patients, respectively. Unilateral transient ILN palsy was reported in 18 (6.8%) cases out of 266 nerves at risk with 2 (0.7%) permanent ILN palsy. No other complications were recorded.

Follow‐up was completed for all patients after a median time of 34 (17–54) months. RAI was administered in 34 (53.9%) patients who underwent immediate CT plus b‐CND (24 cases) or delayed CT (10 cases). No residual or recurrent disease was observed.

**FIGURE 2:**
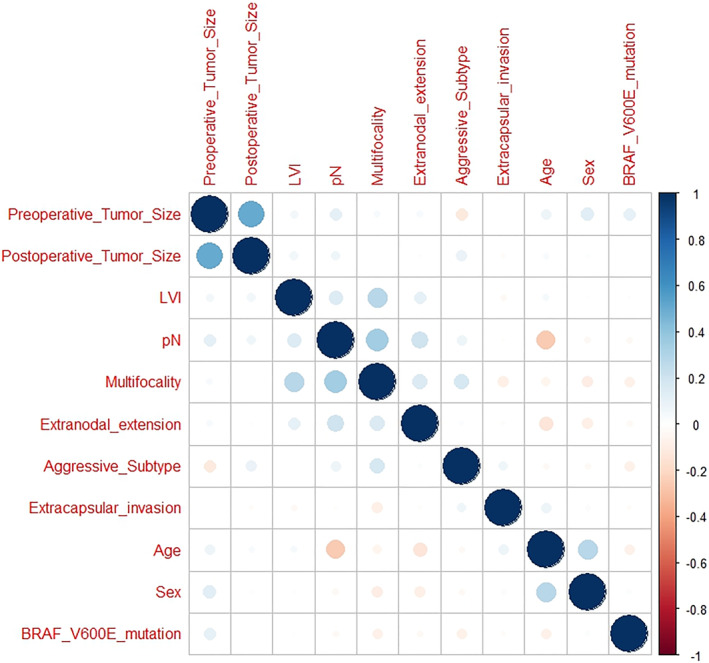
Plot of the correlation matrix: The variables with the positive correlation are highlighted in blue and those with the negative correlation are highlighted in red. The size of the spheres indicates the degree of the correlation. The larger the size of the sphere, the stronger the degree of the correlation; on the other hand, the weaker the degree of the correlation, the smaller the sphere.

**FIGURE 3:**
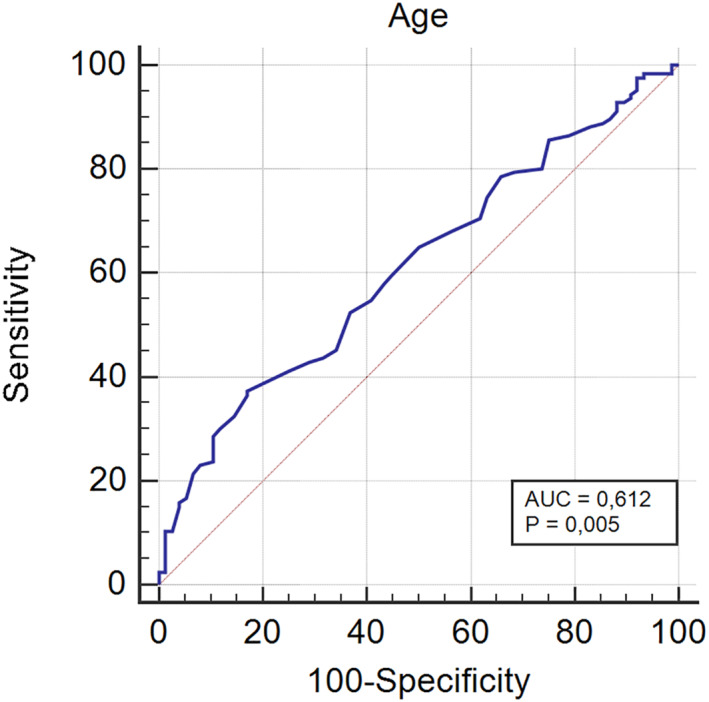
ROC curve. The AUC of the ROC curve for the correlation of age and LNMs was 0.612 (*p* = 0.005); the threshold value for age was set at 35 years (37.3% sensitivity and 82.9% specificity). AUC, area under the curve; LNMs, lymph node metastases; and ROC, receiver operating characteristic.

A comparative analysis between pN0 and pN1a patients was performed to evaluate risk factors for occult central LNMs (Table 3). Univariable analysis was statistically significant for age (*p* < 0.001), LVI (*p* = 0.037), and multifocality (*p* < 0.001). After logistic regression analysis, age (*p* < 0.001) and multifocality (*p* < 0.001) were identified as independent risk factors for LNMs. The plot of the correlation matrix summarizes the correlation between the considered variables (Figure 2). The AUC of the ROC curve for the correlation of age and LNMs was 0.612 (*p* = 0.005) (Figure 3); the threshold value for age was set at 35 years (37.3% sensitivity and 82.9% specificity).

**TABLE 3:** Comparative analysis between pN0 and pN1a patients.

	pN0 (*n* = 127)	pN1a (*n* = 76)	OR (univariable)	OR (multivariable)
Age				
Mean (SD)	43.9 (11.7)	37.5 (12.1)	0.95 (0.93–0.98 and ***p* < 0**.**001**)	0.94 (0.92–0.97 and ***p* < 0**.**001**)
Sex				
F	96 (61.5%)	60 (38.5%)	‐	‐
M	31 (66.0%)	16 (34.0%)	0.83 (0.41–1.62 and *p* = 0.583)	1.36 (0.61–3.00 and *p* = 0.448)
Preoperative tumor size				
Mean (SD)	5.4 (1.9)	5.8 (1.7)	1.14 (0.97–1.34 and *p* = 0.109)	1.16 (0.94–1.43 and *p* = 0.163)
Postoperative tumor size				
Mean (SD)	6.3 (2.1)	6.5 (1.9)	1.07 (0.92–1.23 and *p* = 0.373)	1.02 (0.85–1.24 and *p* = 0.800)
Aggressive subtype				
No	111 (64.2%)	62 (35.8%)	‐	‐
Yes	16 (53.3%)	14 (46.7%)	1.57 (0.71–3.43 and *p* = 0.260)	1.20 (0.48–2.95 and *p* = 0.689)
Extracapsular invasion				
No	125 (62.5%)	75 (37.5%)	‐	‐
Yes	2 (66.7%)	1 (33.3%)	0.83 (0.04–8.84 and *p* = 0.882)	2.21 (0.10–26.29 and *p* = 0.539)
LVI				
No	76 (69.1%)	34 (30.9%)	‐	‐
Yes	51 (54.8%)	42 (45.2%)	1.84 (1.04–3.29 and ***p* =** **0**.**037**)	1.39 (0.71–2.69 and *p* = 0.336)
Multifocality				
No	100 (74.6%)	34 (25.4%)	‐	‐
Yes	27 (39.1%)	42 (60.9%)	4.58 (2.48–8.61 and ***p* < 0**.**001**)	4.44 (2.23–9.12 and ***p* < 0**.**001**)
BRAF‐V600E mutation				
No	122 (62.2%)	74 (37.8%)	‐	‐
Yes	5 (71.4%)	2 (28.6%)	0.66 (0.09–3.14 and *p* = 0.624)	0.59 (0.08–3.21 and *p* = 0.562)

*Note*: Bold values indicate the significative results.

Abbreviations: LVI, lymphovascular invasion; OR, odds ratio; and SD, standard deviation.

Moreover, preoperative characteristics were compared between patients with and without aggressive biological features detected after definitive histopathology (Table 4).

**TABLE 4:** Comparative analysis between patients with and without biological aggressive features.

	0 (*n* = 56)	≥1 (*n* = 147)	OR (univariable)	OR (multivariable)
Age				
Mean (SD)	43.3 (10.6)	40.8 (12.8)	0.98 (0.96–1.01 and *p* = 0.204)	0.98 (0.95–1.01 and *p* = 0.143)
Sex				
F	44 (28.2%)	112 (71.8%)	‐	‐
M	12 (25.5%)	35 (74.5%)	1.15 (0.56–2.48 and *p* = 0.719)	1.29 (0.60–2.94 and *p* = 0.525)
Preoperative tumor size				
Mean (SD)	5.4 (1.9)	5.6 (1.8)	1.09 (0.92–1.29 and *p* = 0.345)	1.09 (0.92–1.30 and *p* = 0.334)

Abbreviations: OR, odds ratio and SD, standard deviation.

## 4 DISCUSSION

This study challenges the pervasive assumption that unifocal cN0 PTMC always represents an indolent disease. Seventy‐six (37.4%) out of all patients had LNMs, which were not evident on preoperative ultrasound, and only a minority (27.6%) of patients did not present biological aggressive features after pathologic evaluation. Our study also highlights a close correlation among LNMs, young age, multifocality, and LVI. These results may explain the skepticism regarding the wide application of nonsurgical treatments in these patients' cohort.

Despite multifocal disease and isolated BRAF‐V600E mutation have been advocated as “appropriate” categories for AS,^33^ other authors reported these factors as high‐risk features for nodal metastases and recurrence.^34^, ^35^

On the other hand, LVI has been identified as a risk factor for undertreatment as it is not easily detectable during AS.^9^, ^36^ Indeed, LVI‐related shorter disease‐free survival (DFS) and overall survival (OS) rely on the prevalence of LVI in aggressive variants of PTC.^37^, ^38^, ^39^

Recent studies confirmed that LNMs as a potential marker of PTMC aggressive behavior and a risk factor for recurrence, distant metastases, reduced survival, and higher morbidity.^16^, ^19^, ^40^, ^41^ At the time of surgery, central LNMs were observed in up to 42% of PTMC patients in different studies despite a negative preoperative imaging evaluation.^42^, ^43^, ^44^, ^45^, ^46^, ^47^ Nevertheless, clinical significance of the nodal status in PTMC still remains a matter of discussion.^16^, ^19^, ^40^, ^41^ Detractors of p‐CND emphasize the increased risk of postoperative complications underestimating the prognostic and therapeutic role.^48^, ^49^

As a matter of fact, to date, most studies analyze an oncologic outcome after TT with and without CND rather than focusing on the prognostic impact of CND associated with TL.^7^, ^50^, ^51^ The extensive use of RAI in the adjuvant setting in the past years may represent a further bias.^7^, ^32^, ^50^

Many authors highlighted the correlation between nodal status and other biologic aggressive features: Cheng et al.^9^ showed that LNMs, together with age, tumor size, and extrathyroidal extension, relate to LVI. As LVI was significantly associated with higher risk of distant metastases, it represented an independent factor for incomplete response to therapy among patients eligible for TL.^36^, ^52^ Other authors also advocated male sex, younger age, and subcapsular tumor localization as a risk factor for LNMs.^34^, ^53^, ^54^

Our analysis showed similar results. In detail, younger age (*p* < 0.001), LVI (*p* = 0.037), and multifocality (*p* < 0.001) are related with pN+. After multivariable analysis, age (*p* < 0.001) and multifocality (*p* < 0.001) were confirmed independent risk factors for LNMs. ROC curve analysis was used to correlate age and nodal status, with the threshold value for age set at 35 years (37.3% sensitivity and 82.9% specificity). However, no cut‐off beyond which nodal involvement could be safely excluded was identified.

Furthermore, no preoperative parameter was able to predict the presence of LMNs nor further biological aggressive features in these patients' cohort, though N+ status has been more frequently reported in younger population.

In our experience, only 56 out of 203 patients were free from biologic aggressive features after definitive histopathology and potential candidates for conservative management. Unfortunately, the correct surgical treatment for PTMC could only be elicited postsurgery.

Of note, nodal status is the only biologic aggressive feature which can be assessed through FSE before definitive histology. In this context, since 2014, we proposed and validated i‐CND FSE for more accurate risk stratification and subsequent therapeutic modulation of unifocal cN0 PTCs.^24^, ^55^ Our results showed i‐CND FSE sensitivity, specificity, and accuracy of 78%, 95%, and 82%, respectively, in detecting occult LNMs, thus allowing the intraoperative identification of those patients who may benefit the most from more extensive surgery.

The sensitivity of current ultrasound methods for detecting central compartment LNMs ranges only from 23% to 53.2%.^56^, ^57^ Although not with unanimous agreement, most authors underlined that regional LNMs are associated with increased local recurrence rates and reduced survival.^58^, ^59^ One publication reported that CND may decrease the false‐positive detection of skip metastases in PTC.^60^ Adequate lymphadenectomy may ensure no residual LNM and reduce secondary surgery rates.^19^, ^60^ In this view, our results show that in 22.9% of patients, FSE modified the extend of surgical dissection.

In high‐volume referral centers and in selected cases, p‐CND is associated with both lower number of local relapses and low risk of complications as reported in several recent meta‐analyses.^61^, ^62^, ^63^

Even though our reported hypoparathyroidism rates (31.7% transient and 6.3% permanent) are relatively high in our selected population, CND is to be considered therapeutic rather than prophylactic given the presence of proven LNMs. In this perspective, our results are also in line with data reported after TT plus therapeutic CND.^64^ Wang et al.^65^ meta‐analysis showed comparable rates: 0%–9.8% transient ILN palsy, 0.4%–4.0% permanent ILN palsy, 7.4%–68.9% transient hypoparathyroidism, and 0%–8.1% permanent hypoparathyroidism.

The present study has the merit to correlate LNMs rate, biological aggressive features, and modulation of therapeutic strategy in patients with unifocal cN0 PTMC. Furthermore, all patients underwent surgical treatment in the same high‐volume referral center based on the same protocol.

The major limitations are the retrospective design, the relatively limited sample size, and the lack of a control group with conservative management.

Nonsurgical treatments for low‐risk PTMC showed to be safe especially in Asian population.^66^ Similar experiences have been reported in other countries even though cultural behaviors limited their global adoption.^67^, ^68^, ^69^, ^70^

It remains to define whether a randomized study comparing surgical and noninterventional management would be ethically acceptable, as this would imply offering an inadequate oncological treatment to up to 72.4% of patients according to our results. A randomized trial would likely require more than 10 years follow‐up to correctly evaluate the oncologic impact of occult subclinical LNMs on OS and DFS.

Furthermore, at the beginning of our application of FSE, many patients expressed their preference for a more radical treatment most probably due to European culture reasons. Due to this, most of the patients were recruited in the latter part of the study, thus explaining the short follow‐up.

Molecular analysis was limited to evaluation of BRAF‐V600E mutation.

In conclusion, although robust evidence showed that most PTMC are eligible to conservative management, a non‐negligible rate of patients may present one or more biologically aggressive features including nodal involvement. Nonsurgical treatments need careful evaluation to avoid undertreatment especially in the young population. Hopefully, future randomized studies with longer follow‐up times will draw definitive conclusions concerning this controversial topic.

FSE may represent the currently available most strategic tool to intraoperatively evaluate nodal status to properly tailor surgical extent, thus balancing oncologic benefit with potential postoperative complications.

## AUTHOR CONTRIBUTIONS

**Francesco Pennestrì**: Conceptualization; formal analysis; investigation; methodology; supervision; visualization; writing–original draft; writing–review & editing. **Priscilla Francesca Procopio**: Conceptualization; methodology; visualization; writing–original draft; writing–review & editing. **Antonio Laurino**: Conceptualization; data curation; investigation; validation; visualization; writing–original draft; writing–review & editing. **Annamaria Martullo**: Data curation; investigation; methodology. **Gloria Santoro**: Formal analysis; methodology; validation. **Pierpaolo Gallucci**: Data curation; investigation; visualization. **Francesca Prioli**: Data curation; investigation; visualization. **Luca Sessa**: Data curation; validation; visualization. **Esther Diana Rossi**: Investigation; supervision; validation. **Alfredo Pontecorvi**: Methodology; validation. **Carmela De Crea**: Conceptualization; supervision; writing–review and editing. **Marco Raffaelli**: Conceptualization; supervision; validation; writing–review and editing.

## CONFLICT OF INTEREST STATEMENT

Marco Raffaelli has a consultant agreement with Medtronic, AB Medica, and Intuitive Surgical. Priscilla Francesca Procopio, Antonio Laurino, Francesco Pennestrì, and Pierpaolo Gallucci have a consultant agreement with Medtronic. Annamaria Martullo, Gloria Santoro, Francesca Prioli, Luca Sessa, Esther Diana Rossi, Alfredo Pontecorvi, and Carmela De Crea declare that they have no conflicts of interest.

## ETHICS STATEMENT

The study was approved by the Ethical Committee of our Center (ID 6584). All procedures performed in studies involving human participants were in accordance with the ethical standards of the institutional and/or national research committee and with the 1964 Helsinki Declaration and its later amendments or comparable ethical standards.

## INFORMED CONSENT

Informed consent was obtained from all individual participants for whom identifying information is included in this article.

## RESEARCH INVOLVING HUMAN PARTICIPANTS AND/OR ANIMALS

This article does not contain any studies with animals performed by any of the authors.
